# Synthesis and Larvicidal Activity against *Culex pipiens pallens* of New Triazole Derivatives of Phrymarolin from *Phryma leptostachya* L.

**DOI:** 10.3390/ijms141224064

**Published:** 2013-12-10

**Authors:** Ji-Wen Zhang, Zhan Hu, Peng Gao, Jun-Ru Wang, Zhao-Nong Hu, Wen-Jun Wu

**Affiliations:** 1 College of Sciences, Northwest A&F University, Yangling 712100, China; E-Mails: nwzjw@nwsuaf.edu.cn (J.-W.Z.); huzhan@aliyun.com (Z.H.); wangjr07@163.com (J.-R.W.); nwzjw@163.com (W.-J.W.); 2 Institute of Pesticide Science, Northwest A&F University, Yangling 712100, China; 3 School of Life Science and Engineering, Southwest Jiaotong University, Chengdu 611756, China; E-Mail: gaopeng1960@foxmail.com

**Keywords:** *Phryma leptostachya* L., triazole, larvicidal activity, *Culex pipiens pallens*

## Abstract

Twelve new triazole derivatives of Phrymarolin were prepared from Phrymarolin I and the structures of all the derivatives were fully characterized by ^1^H-NMR, ^13^C-NMR and MS spectral data analyses. Larvicidal activities against 4rd instar larvae of *Culex pipiens pallens* of these Phrymarolin analogues were assayed. Although the triazole derivatives of Phrymarolin showed certain larvicidal activity, they showed lower activity than Phrymarolin I. The typical non-natural groups triazole substituents reduced the larvicidal activity of Phrymarolin derivatives.

## 1. Introduction

**Figure 1.**
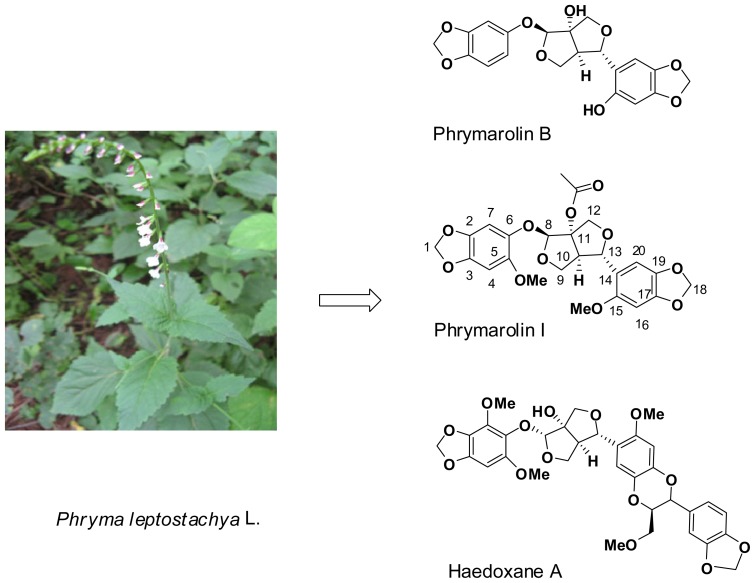
*Phryma leptostachya* L. and its characteristic lignans.

Mosquitoes are significant public health pests for humans and domesticated animals, responsible for spreading serious vector-borne diseases like malaria, filariasis, epidemic encephalitis, yellow fever, dengue, *etc*. A significant increase in the quality of life over the recent decades stems from the successful control of mosquitoes with synthetic chemical pesticides [1]. However, the rising resistance to available synthetic pesticides, combined with their adverse side effects to humans and to the environment, is driving us to search for new alternative insecticides. Investigation of natural products with insecticidal activities from plant may lead to find novel insecticides as the prominent examples of pyrethroids. The herbaceous perennial plant, *Phryma leptostachya* L., is widely distributed in the Himalayas, temperate Asia and northeast America, and has been traditionally used as a natural insecticide in East Asia [2–7]. As part of an ongoing effort to identify potential pesticide molecules derived from natural products it was shown that lignans including Haedoxane A, Phrymarolin B and Phrymarolins I are the main active components of *P. leptostachya* L. (Figure 1) [8–10]. In our previous study [11–13], we found that some of the C-11 ether derivatives and dehydroxy Phrymarolin showed much higher activity than Phrymarolins I. We were interested in making analogues with a triazole substituent linked to C-11, since triazole is a common group to a well-known class of pesticides. In this paper, the synthesis and larvicidal activity against *Culex pipiens pallens* that is the most common mosquito in houses of Northern China and the primary vector of filariasis and epidemic encephalitis of novel triazole derivatives of Phrymarolin I will be described.

## 2. Results and Discussion

**Scheme 1.**
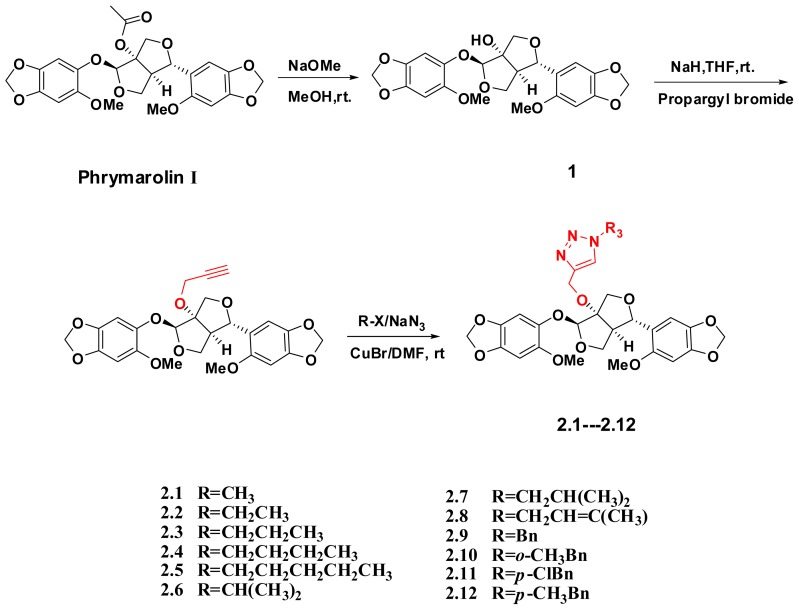
The synthetic route of new triazole derivatives of Phrymarolin.

Phrymarolins I derivatives, were prepared according to the method outlined in Scheme 1 [14]. All the structures of these derivatives were established on the basis of NMR and ESI-MS spectroscopic analysis. Phrymarolin I was isolated from the methanol extration of the plant *Phryma leptostachya* L. showed a [M + Na]^+^ ion at *m*/*z* 511 and [M + H]^+^ ion at *m*/*z* 489 in the ESI-MS, corresponding to the molecular formula C_24_H_24_O_11_. Deacetylphrymarolins I (compound **1**) was obtained from Phrymarolins I by treatment with MeONa in methanol and compound **2** was obtained from compound **1** by treatment with propargyl bromide and NaH in dry THF. The triazole derivatives of Phrymarolin compounds **2.1**–**2.12** were obtained from compound **2** by treatment with according halohyrocarbon and NaN_3_ together with catalytic amount CuBr and column chromatographic purification afforded the target molecules and some experiment data of compounds **2.1**–**2.12** can be found in Table 1.

4rd instar larvae of *Culex pipiens pallens* were used as tested insects and the concentrations of the tested compounds were 20 ppm. Mortality was recorded after 12 h. The larvicidal activity results can be found in Table 2 and the results suggested that the triazole groups installed to C-11 position in phrymarolins slightly reduce the larvicidal activity of phrymarolin derivatives.

## 3. Experimental Section

### 3.1. General

Melting points were measured on an electrothermal digital apparatus made in Beijing and were uncorrected. The ^1^H-NMR (500 MHz) and ^13^C-NMR (125 MHz) were obtained on a Bruker AM-500 FT-NMR spectrometer (Bruker, Switzerland) with CDCl_3_ as the solvent and TMS as the internal standard. MS were recorded under ESI conditions using a Thermo LCQ Fleet instrument (Thermo, Waltham, MA, USA). Optical rotation was measured by Rudolph Autopol II (Rudolph Research Analytical, Hackettstown, NJ, USA). Analytical thin layer chromatography (TLC) was carried out on precoated plates (silica gel), and spots were visualized with UV and I_2_. Yields were not optimized. Solvents were dried by standard methods. The title compounds were synthesized under an argon atmosphere.

### 3.2. Synthesis of Derivatives of Phrymarolins I

Compound **1**: 4.88 g (10 mmol) Phrymarolins I was dissolved in 100 mL MeOH in a 250 mL three-neck-bottle and 1.08 g (20 mmol) MeONa was added into the reaction bottle and stirred over night. When the reaction was completed (checked by TLC), 20 mL saturated ammonium chloride was added to quench the reaction, then 100 mL water was added and extracted with ethyl acetate (150 mL × 3). The ethyl acetate layers were combined and washed with 50 mL water and 30 mL saturated sodium chloride, dried over anhydrous sodium sulfate and separated by column chromatography (silica gel, 200–300 mesh) with a gradient of petroleum ether (60–90 °C) and ethyl acetate as eluent to yield compound **1** as white powder. *M.P.*: 58–60 °C, ESI-MS (*m*/*z*): 471 [M + Na]^+^. ${\left[\alpha \right]}_{\text{D}}^{28}$: +66.37. ^1^H-NMR (CDCl_3_, 500 MHz), δ 7.15 (s, 1H, Ph–CH), 6.79 (s, 1H, Ph–CH), 6.55 (s, 1H, Ph–CH), 6.50 (s, 1H, Ph–CH), 5.91 (m, 2H, CH_2_), 5.89 (m, 2H, CH_2_), 5.18 (s, 1H, CH), 4.93 (d, 1H, *J* = 6.5 Hz, CH), 4.44 (dd, 1H, *J* = 7, 10 Hz, 9-CH_2_–H_B_), 4.31 (d, 1H, *J* = 10 Hz, 12-CH_2_–H_B_), 4.08 (dd, 1H, *J* = 2, 10 Hz, 9-CH_2_–H_A_), 3.76 (s, 3H, OMe), 3.75 (s, 3H, OMe), 3.73 (d, 1H, *J* = 10 Hz, 12-CH_2_–H_A_), 2.59 (m, 1H, CH); ^13^C-NMR (CDCl_3_, 125 MHz), δ 151.13 (C), 147.23 (C), 145.59 (C), 143.54 (C), 141.33 (C), 140.76 (C), 139.54 (C), 122.27 (C), 106.46 (CH), 105.75 (CH), 102.79 (CH), 101.34 (CH_2_), 101.15 (CH_2_), 96.71 (CH), 94.14 (CH), 92.27 (C), 84.08 (CH), 77.38 (CH_2_), 71.38 (CH_2_), 58.12 (CH), 56.83 (OMe), 56.18 (OMe).

Compound **2**: NaH (50 mg, >1 mmol) was added to a solution of compound **1** (92 mg, 0.2 mmol) in dry tetrahydrofuran (50 mL) at room temperature and stirred for 30 min. Propargyl bromide (0.2 mL) was dropped into the mixture and stirred overnight at room temperature. When the reaction was completed (checked by TLC), the reaction mixture was poured into 50 mL saturated ammonium chloride and extracted with ethyl acetate (50 mL × 3). The ethyl acetate layers were combined and washed with 30 mL water and 5 mL saturated sodium chloride, dried over anhydrous sodium sulfate and separated by column chromatography (silica gel, 200–300 mesh) with a gradient of petroleum ether (60–90 °C) and ethyl acetate as eluent to yield compound **2** as white powder. ESI-MS (*m*/*z*): 507 [M + Na]^+^, ^1^H-NMR (CDCl_3_, 500 MHz), δ 7.13 (s, 1H, Ph–CH), 6.76 (s, 1H, Ph–CH), 6.56 (s, 1H, Ph–CH), 6.50 (s, 1H, Ph–CH), 5.91 (s, 2H, CH_2_), 5.88 (m, 2H, CH_2_), 5.31 (s, 1H, CH), 4.88 (d, 1H, *J* = 6.5 Hz, CH), 4.66 (d, 1H, *J* = 10 Hz, 12-CH_2_–H_B_), 4.44 (dd, 1H, *J* = 7, 9 Hz, 9-CH_2_–H_B_), 4.06 (dd, 1H, *J* = 2, 9 Hz, 9-CH_2_–H_A_), 3.76 (s, 3H, OMe), 3.73 (s, 3H, OMe), 3.53 (d, 1H, *J* = 10 Hz, 12-CH_2_–H_A_), 2.82 (m, 1H, CH); R–H: 4.52 (dd, 1H, *J* = 15, 5 Hz, CH_2_–H_B_), 4.40 (dd, 1H, *J* = 2, 5 Hz, CH_2_–H_A_), 2.44 (t, 1H, *J* = 2.5 Hz, Propargyl–CH); ^13^C-NMR (CDCl_3_, 125 MHz), δ 151.21 (C), 147.28 (C), 145.95 (C), 143.38 (C), 141.29 (C), 140.73 (C), 139.44 (C), 121.95 (C), 106.41 (CH), 103.75 (CH), 103.45 (CH), 101.27 (CH_2_), 101.16 (CH_2_), 97.81 (C), 96.15 (CH), 94.16 (CH), 83.98 (CH), 74.12 (CH_2_), 70.48 (CH_2_), 56.93 (OMe), 56.18 (OMe), 55.95 (CH); R–C: 80.78 (C), 74.26 (CH), 55.03 (CH_2_).

Compound **2.1**: NaN_3_ 4.9 mg (76 mmol) was added to a 25 mL eggplant type flask with 10 mL dry dimethylformamide (DMF) and 0.2 mL (3 mmol) methyl iodide at room temperature. After stirred for 10 min 44.6 mg (0.1 mmol) compound **2** and catalytic amount copper (I) bromide (CuBr) were added into the mixture and stirred overnight at room temperature under a argon atmosphere. When the reaction was completed (checked by TLC), the reaction mixture was poured into 50 mL saturated ammonium chloride and extracted with dichloromethane (50 mL × 3). The DCM layers were combined and washed with 30 mL water and 5 mL saturated sodium chloride, dried over anhydrous sodium sulfate and separated by column chromatography(silica gel, 200–300 mesh) with a gradient of petroleum ether (60–90 °C) and ethyl acetate as eluent to yield compound **2.1** as white needle solid. ESI-MS (*m*/*z*): 524 [M + Na]^+^, ^1^H-NMR (CDCl_3_, 500 MHz), δ 7.05 (s, 1H, Ph–CH), 6.75 (s, 1H, Ph–CH), 6.56 (s, 1H, Ph–CH), 6.50 (s, 1H, Ph–CH), 5.91 (s, 2H, CH_2_), 5.89 (m, 2H, CH_2_), 5.38 (s, 1H, CH), 4.90 (d, 1H, *J* = 6.5 Hz, CH), 4.59 (d, 1H, *J* = 10 Hz, 12-CH_2_–H_B_), 4.47 (dd, 1H, *J* = 7, 9 Hz, 9-CH_2_–H_B_), 4.08 (dd, 1H, *J* = 2, 9 Hz, 9-CH_2_–H_A_), 3.76 (s, 3H, OMe), 3.74 (s, 3H, OMe), 3.59 (d, 1H, *J* = 10 Hz, 12-CH_2_–H_A_), 2.79 (m, 1H, CH); R–H: 7.55 (s, 1H, =CH), 5.06, 4.86 (q, 2H, 12.5 Hz, CH_2_), 4.05 (s, 3H, N–CH_3_); ^13^C-NMR (CDCl_3_, 125 MHz), δ 151.22 (C), 147.29 (C), 146.00 (C), 143.52 (C), 141.24 (C), 140.68 (C), 101.16 (CH_2_), 139.14 (C), 121.91 (C), 106.36 (CH), 103.91 (CH), 103.32 (CH), 101.29 (CH_2_), 97.99 (C), 96.02 (CH), 94.17 (CH), 83.88 (CH), 74.06 (CH_2_), 70.39 (CH_2_), 56.89 (OMe), 56.19 (OMe), 56.09 (CH); R–C: 146.66 (C), 123.56 (CH), 61.50 (CH_2_), 36.61 (N–CH_3_).

Compound **2.2**–**2.12** were prepared in the same way.

Compound **2.2**. white needle solid; ESI-MS (*m*/*z*): 578[M + Na]^+^, ^1^H-NMR (CDCl_3_, 500 MHz), δ 7.06 (s, 1H, Ph–CH), 6.76 (s, 1H, Ph–CH), 6.56 (s, 1H, Ph–CH), 6.50 (s, 1H, Ph–CH), 5.91 (s, 2H, CH_2_), 5.89 (m, 2H, CH_2_), 5.38 (s, 1H, CH), 4.92 (d, 1H, *J* = 6.5 Hz, CH), 4.60 (d, 1H, *J* = 10 Hz, 12-CH_2_–H_B_), 4.47 (dd, 1H, *J* = 7, 9 Hz, 9-CH_2_–H_B_), 4.09 (dd, 1H, *J* = 2, 9 Hz, 9-CH_2_–H_A_), 3.76 (s, 3H, OMe), 3.74 (s, 3H, OMe), 3.60 (d, 1H, *J* = 10 Hz, 12-CH_2_–H_A_), 2.81 (m, 1H, CH); R–H: 7.57 (s, 1H, =CH), 5.06, 4.86 (dd, 2H, 12.5 Hz, CH_2_), 4.36 (q, 2H, 6.5 Hz, N–CH_2_), 1.53 (t, 6.5 Hz, 3H, CH_3_); ^13^C-NMR (CDCl_3_, 125 MHz), δ 151.22 (C), 147.29 (C), 145.99 (C), 143.49 (C), 141.24 (C), 140.68 (C), 139.20 (C), 121.85 (C), 106.37 (CH), 103.86 (CH), 103.34 (CH), 101.28 (CH_2_), 101.15 (CH_2_), 97.93 (C), 96.06 (CH), 94.17 (CH), 83.89 (CH), 74.04 (CH_2_), 70.45 (CH_2_), 56.89 (OMe), 56.19 (OMe), 56.03 (CH); R–C: 146.38 (C), 121.97 (CH), 61.55 (CH_2_), 45.22 (N–CH_2_), 15.43 (CH_3_).

Compound **2.3**. yellow solid; ESI-MS (*m*/*z*): 592[M + Na]^+^, ^1^H-NMR (CDCl_3_, 500 MHz), δ 7.06 (s, 1H, Ph–CH), 6.75 (s, 1H, Ph–CH), 6.56 (s, 1H, Ph–CH), 6.50 (s, 1H, Ph–CH), 5.91 (s, 2H, CH_2_), 5.89 (m, 2H, CH_2_), 5.38 (s, 1H, CH), 4.92 (d, 1H, *J* = 6.5 Hz, CH), 4.60 (d, 1H, *J* = 10 Hz, 12-CH_2_–H_B_), 4.48 (dd, 1H, *J* = 7, 9 Hz, 9-CH_2_–H_B_), 4.09 (dd, 1H, *J* = 2, 9 Hz, 9-CH_2_–H_A_), 3.76 (s, 3H, OMe), 3.74 (s, 3H, OMe), 3.60 (d, 1H, *J* = 10 Hz, 12-CH_2_–H_A_), 2.81 (m, 1H, CH); R–H: 7.55 (s, 1H, =CH), 5.07, 4.86 (dd, 2H, 12.5 Hz, CH_2_), 4.29 (t, 2H, 6.5 Hz, N–CH_2_), 1.90 (m, 2H, CH_2_), 0.93 (t, 3H, 6.5 Hz, CH_3_); ^13^C-NMR (CDCl_3_, 125 MHz), δ 151.22 (C), 147.28 (C), 146.00 (C), 143.48 (C), 141.23 (C), 140.68 (C), 139.21 (C), 121.97 (C), 106.36 (CH), 103.87 (CH), 103.34 (CH), 101.28 (CH_2_), 101.15 (CH_2_), 97.94 (C), 96.06 (CH), 94.17 (CH), 83.89 (CH), 74.05 (CH_2_), 70.45 (CH_2_), 56.93 (OMe), 56.18 (OMe), 56.04 (CH); R–C: 146.26 (C), 122.12 (CH), 61.58 (CH_2_), 51.89 (N–CH_2_), 23.67 (CH_2_), 11.08 (CH_3_).

Compound **2.4**. yellow solid; ESI-MS (*m*/*z*): 606 [M + Na]^+^, ^1^H-NMR (CDCl_3_, 500 MHz), δ 7.07 (s, 1H, Ph–CH), 6.75 (s, 1H, Ph–CH), 6.56 (s, 1H, Ph–CH), 6.50 (s, 1H, Ph–CH), 5.91 (s, 2H, CH_2_), 5.89 (m, 2H, CH_2_), 5.38 (s, 1H, CH), 4.92 (d, 1H, *J* = 6.5 Hz, CH), 4.60 (d, 1H, *J* = 10 Hz, 12-CH_2_–H_B_), 4.48 (dd, 1H, *J* = 7, 9 Hz, 9-CH_2_–H_B_), 4.09 (dd, 1H, *J* = 2, 9 Hz, 9-CH_2_–H_A_), 3.76 (s, 3H, OMe), 3.74 (s, 3H, OMe), 3.59 (d, 1H, *J* = 10 Hz, 12-CH_2_–H_A_), 2.81 (m, 1H, CH); R–H: 7.57 (s, 1H, =CH), 5.06, 4.86 (dd, 2H, 12.5 Hz, CH_2_), 4.31 (t, 2H, 6.5 Hz, N–CH_2_), 1.86 (m, 2H, CH_2_), 1.33 (m, 2H, CH_2_), 0.93 (t, 3H, 6.5 Hz, CH_3_); ^13^C-NMR (CDCl_3_, 125 MHz), δ 151.22 (C), 147.28 (C), 146.01 (C), 143.49 (C), 141.24 (C), 140.68 (C), 139.21 (C), 121.98 (C), 106.37 (CH), 103.88 (CH), 103.34 (CH), 101.28 (CH_2_), 101.15 (CH_2_), 97.95 (C), 96.07 (CH), 94.17 (CH), 83.89 (CH), 74.03 (CH_2_), 70.45 (CH_2_), 56.94 (OMe), 56.19 (OMe), 56.06 (CH); R–C: 146.26 (C), 122.10 (CH), 61.57 (CH_2_), 50.06 (N–CH_2_), 32.21 (CH_2_), 19.73 (CH_2_), 13.45 (CH_3_).

Compound **2.5**. yellow solid; ESI-MS (*m*/*z*): 620 [M + Na]^+^, ^1^H-NMR (CDCl_3_, 500 MHz), δ 7.07 (s, 1H, Ph–CH), 6.75 (s, 1H, Ph–CH), 6.56 (s, 1H, Ph–CH), 6.50 (s, 1H, Ph–CH), 5.91 (s, 2H, CH_2_), 5.89 (m, 2H, CH_2_), 5.38 (s, 1H, CH), 4.91 (d, 1H, *J* = 6.5 Hz, CH), 4.60 (d, 1H, *J* = 10 Hz, 12-CH_2_–H_B_), 4.48 (dd, 1H, *J* = 7, 9 Hz, 9-CH_2_–H_B_), 4.09 (dd, 1H, *J* = 2, 9 Hz, 9-CH_2_–H_A_), 3.76 (s, 3H, OMe), 3.74 (s, 3H, OMe), 3.60 (d, 1H, *J* = 10 Hz, 12-CH_2_–H_A_), 2.81 (m, 1H, CH); R–H: 7.56 (s, 1H, =CH), 5.06, 4.86 (dd, 2H, 12.5 Hz, CH_2_), 4.29 (t, 2H, 6.5 Hz, N–CH_2_), 1.88 (m, 2H, CH_2_), 1.32 (m, 4H, CH_2_ × 2), 0.88 (t, 3H, 6.5 Hz, CH_3_); ^13^C-NMR (CDCl_3_, 125 MHz), δ 151.23 (C), 147.29 (C), 146.01 (C), 143.49 (C), 141.24 (C), 140.69 (C), 139.19 (C), 121.97 (C), 106.38 (CH), 103.88 (CH), 103.33 (CH), 101.28 (CH_2_), 101.15 (CH_2_), 97.95 (C), 96.07 (CH), 94.18 (CH), 83.89 (CH), 74.02 (CH_2_), 70.44 (CH_2_), 56.93 (OMe), 56.19 (OMe), 56.07 (CH); R–C: 146.33 (C), 122.07 (CH), 61.56 (CH_2_), 50.32 (N–CH_2_), 29.95 (CH_2_), 28.61 (CH_2_), 22.08 (CH_2_), 13.84 (CH_3_).

Compound **2.6**. yellow solid; ESI-MS (*m*/*z*): 592 [M + Na]^+^, ^1^H-NMR (CDCl_3_, 500 MHz), δ 7.08 (s, 1H, Ph–CH), 6.76 (s, 1H, Ph–CH), 6.56 (s, 1H, Ph–CH), 6.50 (s, 1H, Ph–CH), 5.91 (s, 2H, CH_2_), 5.89 (m, 2H, CH_2_), 5.38 (s, 1H, CH), 4.93 (d, 1H, *J* = 6.5 Hz, CH), 4.61 (d, 1H, *J* = 10 Hz, 12-CH_2_–H_B_), 4.48 (dd, 1H, *J* = 7, 9 Hz, 9-CH_2_–H_B_), 4.09 (dd, 1H, *J* = 2, 9 Hz, 9-CH_2_–H_A_), 3.76 (s, 3H, OMe), 3.74 (s, 3H, OMe), 3.61 (d, 1H, *J* = 10 Hz, 12-CH_2_–H_A_), 2.83 (m, 1H, CH); R–H: 7.58 (s, 1H, =CH), 5.06, 4.86 (dd, 2H, 12.5 Hz, CH_2_), 4.78 (m, 1H, N–CH), 1.55 (m, 6H, CH_3_ × 2); ^13^C-NMR (CDCl_3_, 125 MHz), δ 151.22 (C), 147.28 (C), 145.99 (C), 143.46 (C), 141.23 (C), 140.60 (C), 139.26 (C), 122.02 (C), 106.37 (CH), 103.80 (CH), 103.33 (CH), 101.28 (CH_2_), 101.15 (CH_2_), 97.86 (C), 96.12 (CH), 94.17 (CH), 83.89 (CH), 74.02 (CH_2_), 70.52 (CH_2_), 56.98 (OMe), 56.19 (OMe), 55.96 (CH); R–C: 142.56 (C), 119.95 (CH), 61.56 (CH_2_), 52.89 (N–CH), 23.01 (CH_3_), 22.97 (CH_3_).

Compound **2.7**. yellow solid; ESI-MS (*m*/*z*): 606 [M + Na]^+^, ^1^H-NMR (CDCl_3_, 500 MHz), δ 7.06 (s, 1H, Ph–CH), 6.75 (s, 1H, Ph–CH), 6.56 (s, 1H, Ph–CH), 6.50 (s, 1H, Ph–CH), 5.91 (s, 2H, CH_2_), 5.89 (m, 2H, CH_2_), 5.38 (s, 1H, CH), 4.91 (d, 1H, *J* = 6.5 Hz, CH), 4.59 (d, 1H, *J* = 10 Hz, 12-CH_2_–H_B_), 4.48 (dd, 1H, *J* = 7, 9 Hz, 9-CH_2_–H_B_), 4.08 (dd, 1H, *J* = 2, 9 Hz, 9-CH_2_–H_A_), 3.76 (s, 3H, OMe), 3.74 (s, 3H, OMe), 3.59 (d, 1H, *J* = 10 Hz, 12-CH_2_–H_A_), 2.81 (m, 1H, CH); R–H: 7.53 (s, 1H, =CH), 5.07, 4.86 (dd, 2H, 12.5 Hz, CH_2_), 4.10 (d, 2H, 7.5 Hz, N–CH_2_), 2.18 (m, 1H, CH), 0.93 (d, 6H, 6.5 Hz, CH_3_ × 2); ^13^C-NMR (CDCl_3_, 125 MHz), δ 151.22 (C), 147.28 (C), 146.01 (C), 143.49 (C), 141.23 (C), 140.68 (C), 139.22 (C), 121.99 (C), 106.36 (CH), 103.89 (CH), 103.36 (CH), 101.28 (CH_2_), 101.15 (CH_2_), 97.97 (C), 96.07 (CH), 94.17 (CH), 83.91 (CH), 74.07 (CH_2_), 70.46 (CH_2_), 56.94 (OMe), 56.18 (OMe), 56.05 (CH); R–C: 146.31 (C), 122.77 (CH), 61.63 (CH_2_), 57.54 (N–CH_2_), 29.66 (CH), 19.87 (CH_3_ × 2).

Compound **2.8**. yellow solid; ESI-MS (*m*/*z*): 617 [M + Na]^+^, ^1^H-NMR (CDCl_3_, 500 MHz), δ 7.05 (s, 1H, Ph–CH), 6.75 (s, 1H, Ph–CH), 6.56 (s, 1H, Ph–CH), 6.50 (s, 1H, Ph–CH), 5.91 (s, 2H, CH_2_), 5.89 (m, 2H, CH_2_), 5.38 (s, 1H, CH), 4.90 (d, 1H, *J* = 6.5 Hz, CH), 4.60 (d, 1H, *J* = 10 Hz, 12-CH_2_–H_B_), 4.47 (dd, 1H, *J* = 7, 9 Hz, 9-CH_2_–H_B_), 4.08 (dd, 1H, *J* = 2, 9 Hz, 9-CH_2_–H_A_), 3.77 (s, 3H, OMe), 3.72 (s, 3H, OMe), 3.58 (d, 1H, *J* = 10 Hz, 12-CH_2_–H_A_), 2.80 (m, 1H, CH); R–H: 7.54 (s, 1H, =CH), 5.40 (m, 1H, =CH), 5.05, 4.85 (dd, 2H, 12.5 Hz, CH_2_), 4.90 (d, 2H, *J* = 6.5 Hz, N–CH_2_), 1.77 (s, 3H, CH_3_), 1.76 (s, 3H, CH_3_); ^13^C-NMR (CDCl_3_, 125 MHz), δ 151.21 (C), 147.28 (C), 146.00 (C), 143.47 (C), 141.26 (C), 140.68 (C), 139.21 (C), 121.95 (C), 106.35 (CH), 103.86 (CH), 103.31 (CH), 101.28 (CH_2_), 101.14 (CH_2_), 97.98 (C), 96.07 (CH), 94.16 (CH), 83.90 (CH), 73.95 (CH_2_), 70.41 (CH_2_), 56.92 (OMe), 56.18 (OMe), 56.12 (CH); R–C: 146.28 (C), 139.56 (C), 121.95 (CH), 117.35 (CH), 61.54 (CH_2_), 54 (CH_2_), 47.93 (N–CH_2_), 25.64 (CH_3_), 18.02 (CH_3_).

Compound **2.9**. yellow solid; ESI-MS (*m*/*z*): 640 [M + Na]^+^, ^1^H-NMR (CDCl_3_, 500 MHz), δ 7.03 (s, 1H, Ph–CH), 6.72 (s, 1H, Ph–CH), 6.52 (s, 1H, Ph–CH), 6.49 (s, 1H, Ph–CH), 5.91 (s, 2H, CH_2_), 5.88 (m, 2H, CH_2_), 5.35 (s, 1H, CH), 4.89 (d, 1H, *J* = 6.5 Hz, CH), 4.57 (d, 1H, *J* = 10 Hz, 12-CH_2_–H_B_), 4.45 (dd, 1H, *J* = 7, 9 Hz, 9-CH_2_–H_B_), 4.07 (dd, 1H, *J* = 2, 9 Hz, 9-CH_2_–H_A_), 3.74 (s, 3H, OMe), 3.62 (s, 3H, OMe), 3.57 (d, 1H, *J* = 10 Hz, 12-CH_2_–H_A_), 2.78 (m, 1H, CH); R–H: 7.50 (s, 1H, =CH), 7.35 (d, 1H, 7.0 Hz, Ph–CH), 7.33 (d, 2H, 7.0 Hz, Ph–CH × 2), 7.25 (d, 2H, 7.0 Hz, Ph–CH × 2), 5.44 (s, 2H, N–CH_2_), 5.01, 4.84 (dd, 2H, 12.5 Hz, CH_2_); ^13^C-NMR (CDCl_3_, 125 MHz), δ 151.20 (C), 147.28 (C), 145.93 (C), 143.44 (C), 141.23 (C), 140.64 (C), 139.17 (C), 121.91 (C), 106.36 (CH), 103.82 (CH), 103.32 (CH), 101.27 (CH_2_), 101.15 (CH_2_), 97.93 (C), 96.04 (CH), 94.15 (CH), 83.95 (CH), 74.03 (CH_2_), 70.42 (CH_2_), 56.82 (OMe), 56.16 (OMe), 55.94 (CH); R–C: 146.66 (C), 134.74 (C), 129.04 (CH × 2), 128.63 (CH), 128.11 (CH × 2), 123.56 (CH), 61.52 (CH_2_), 54.12 (N–CH_2_).

Compound **2.10**. yellow solid; ESI-MS (*m*/*z*): 654 [M + Na]^+^, ^1^H-NMR (CDCl_3_, 500 MHz), δ 7.01 (s, 1H, Ph–CH), 6.71 (s, 1H, Ph–CH), 6.51 (s, 1H, Ph–CH), 6.49 (s, 1H, Ph–CH), 5.91 (s, 2H, CH_2_), 5.89 (m, 2H, CH_2_), 5.34 (s, 1H, CH), 4.87 (d, 1H, *J* = 6.5 Hz, CH), 4.56 (d, 1H, *J* = 10 Hz, 12-CH_2_–H_B_), 4.44 (dd, 1H, *J* = 7, 9 Hz, 9-CH_2_–H_B_), 4.06 (dd, 1H, *J* = 2, 9 Hz, 9-CH_2_–H_A_), 3.74 (s, 3H, OMe), 3.61 (s, 3H, OMe), 3.56 (d, 1H, *J* = 10 Hz, 12-CH_2_–H_A_), 2.77 (m, 1H, CH); R–H: 7.39 (s, 1H, =CH), 7.25 (m, 1H, Ph–CH), 7.17 (m, 2H, Ph–CH × 2), 7.13 (m, 1H, Ph–CH), 5.49 (d, 2H, 7.0 Hz, N–CH_2_), 5.00, 4.83 (dd, 2H, 12.5 Hz, CH_2_), 2.26 (s, 3H, CH_3_); ^13^C-NMR (CDCl_3_, 125 MHz), δ 151.19 (C), 147.27 (C), 145.92 (C), 143.46 (C), 141.24 (C), 140.65 (C), 139.11 (C), 121.88 (C), 106.36 (CH), 103.88 (CH), 103.29 (CH), 101.26 (CH_2_), 101.14 (CH_2_), 97.99 (C), 96.04 (CH), 94.14 (CH), 83.94 (CH), 73.99 (CH_2_), 70.38 (CH_2_), 56.79 (OMe), 56.16 (OMe), 55.99 (CH); R–C: 146.52 (C), 136.87 (C), 132.61 (C), 130.93 (CH), 129.43 (CH), 128.99 (CH), 126.59 (CH), 122.43 (CH), 61.51 (CH_2_), 52.25 (N–CH_2_), 18.96 (CH_3_).

Compound **2.11**. yellow solid; ESI-MS (*m*/*z*): 675 [M + Na]^+^, ^1^H-NMR (CDCl_3_, 500 MHz), δ 7.02 (s, 1H, Ph–CH), 6.72 (s, 1H, Ph–CH), 6.53 (s, 1H, Ph–CH), 6.49 (s, 1H, Ph–CH), 5.91 (s, 2H, CH_2_), 5.89 (m, 2H, CH_2_), 5.35 (s, 1H, CH), 4.89 (d, 1H, *J* = 6.5 Hz, CH), 4.57 (d, 1H, *J* = 10 Hz, 12-CH_2_–H_B_), 4.45 (dd, 1H, *J* = 7, 9 Hz, 9-CH_2_–H_B_), 4.07 (dd, 1H, *J* = 2, 9 Hz, 9-CH_2_–H_A_), 3.74 (s, 3H, OMe), 3.65 (s, 3H, OMe), 3.57 (d, 1H, *J* = 10 Hz, 12-CH_2_–H_A_), 2.77 (m, 1H, CH); R–H: 7.50 (s, 1H, =CH), 5.02, 4.85 (dd, 2H, 12.5 Hz, CH_2_), 5.44 (d, 2H, 7.0 Hz, N–CH_2_), 7.31 (d, 2H, 8.0 Hz, Ph–CH × 2), 7.17 (d, 2H, 8.0 Hz, Ph–CH × 2); ^13^C-NMR (CDCl_3_, 125 MHz), δ 151.21 (C), 147.29 (C), 145.93 (C), 143.51 (C), 141.21 (C), 140.67 (C), 139.07 (C), 121.89 (C), 106.29 (CH), 103.87 (CH), 103.25 (CH), 101.29 (CH_2_), 101.17 (CH_2_), 97.99 (C), 96.01 (CH), 94.17 (CH), 83.91 (CH), 73.95 (CH_2_), 70.42 (CH_2_), 56.82 (OMe), 56.17 (OMe), 56.02 (CH); R–C: 147.03 (C), 134.67 (C), 133.22 (C), 129.40 (2 × CH), 129.25 (2 × CH), 122.51 (CH), 61.49 (CH_2_), 53.34 (N–CH_2_).

Compound **2.12**. yellow solid; ESI-MS (*m*/*z*): 654 [M + Na]^+^, ^1^H-NMR (CDCl_3_, 500 MHz), δ 7.03 (s, 1H, Ph–CH), 6.72 (s, 1H, Ph–CH), 6.52 (s, 1H, Ph–CH), 6.49 (s, 1H, Ph–CH), 5.91 (s, 2H, CH_2_), 5.88 (m, 2H, CH_2_), 5.34 (s, 1H, CH), 4.88 (d, 1H, *J* = 6.5 Hz, CH), 4.57 (d, 1H, *J* = 10 Hz, 12-CH_2_–H_B_), 4.44 (dd, 1H, *J* = 7, 9 Hz, 9-CH_2_–H_B_), 4.07 (dd, 1H, *J* = 2, 9 Hz, 9-CH_2_–H_A_), 3.63 (s, 3H, OMe), 3.74 (s, 3H, OMe), 3.57 (d, 1H, *J* = 10 Hz, 12-CH_2_–H_A_), 2.77 (m, 1H, CH); R–H: 7.48 (s, 1H, =CH), 7.14 (s, 4H, Ph–CH × 4), 5.42 (s, 2H, N–CH_2_), 5.01, 4.83 (dd, 2H, 12.5 Hz, CH_2_), 2.33 (s, 3H, CH_3_); ^13^C-NMR (CDCl_3_, 125 MHz), δ 151.19 (C), 147.27 (C), 145.94 (C), 143.43 (C), 141.23 (C), 140.64 (C), 139.18 (C), 121.92 (C), 106.36 (CH), 103.83 (CH), 103.32 (CH), 101.26 (CH_2_), 101.14 (CH_2_), 97.92 (C), 96.03 (CH), 94.14 (CH), 83.94 (CH), 74.02 (CH_2_), 70.41 (CH_2_), 56.82 (OMe), 55.95 (CH), 56.16 (OMe); R–C: 146.66 (C), 138.51 (C), 131.70 (C), 129.68 (CH × 2), 128.14 (CH × 2), 122.45 (CH), 61.50 (CH_2_), 53.91 (N–CH_2_), 21.14 (CH_3_).

### 3.3. Bioassay for Larvicidal Activities

Larvicidal activities against 4rd instar larvae of *Culex pipiens pallens* of these Phrymarolin analogues were assayed. *Culex pipiens pallens* was continuously maintained in our laboratory without exposure to any insecticide. The larvae of *Culex pipiens pallens* were fed a diet of brewers’ yeast, dog biscuits, and algae collected from ponds in a ratio of 3:1:1 at 27 ± 2 °C and 75%–85% relative humidity under a 12 L:12 D photoperiod. Adult mosquitoes were maintained on a 10% sucrose solution and blood from a live mouse. The larvicidal bioassay followed the World Health Organization standard protocols with suitable modifications. The test samples at the concentrations of 20 ppm were transferred into 24-well-cell-culture-plate (acetone and Phrymarolins I were used as blank and positive controls, choose 20 ppm as the test concentration according to the previous study in our group). Twenty four early fourth instar larvae of *Culex pipiens pallens* were separately introduced into different wells of 24-well-cell-culture-plate. Mortality rate was recorded after 12 h of post exposure. Dead larvae were identified when they failed to move after probing with a needle in the siphon or cervical region. The experiments were replicated thrice on three different days [13].

## 4. Conclusions

In conclusion, 12 novel triazole derivatives of Phrymarolin were semisynthesized using Phrymarolins I from *Phryma leptostachya* L. as a starting material. Although the triazole derivatives of Phrymarolin showed certain larvicidal activity against 4rd instar larvae of *Culex pipiens pallens*. They showed lower activity than Phrymarolin I. The structrue-activity relationship studied results suggested that triazole groups induced to C-11 position in phrymarolins may reduce the larvicidal activity of phrymarolin derivatives, especially large groups induced to C-11 position in phrymarolins.

## Figures and Tables

**Table 1.** Experiment data of compounds **2.1**–**2.12**.

Compounds	Formula	ESI-MS (*m*/*z*)	M.P./°C	_${\left[\alpha \right]}_{\text{D}}^{27}$_
**2.1**	C_26_H_27_N_3_O_10_	564[M + Na]^+^	146–148	+64.155
**2.2**	C_27_H_29_N_3_O_10_	578[M + Na]^+^	52–54	+39.208
**2.3**	C_28_H_31_N_3_O_10_	592[M + Na]^+^	62–64	+33.246
**2.4**	C_29_H_33_N_3_O_10_	606[M + Na]^+^	58–60	+50.311
**2.5**	C_30_H_35_N_3_O_10_	620[M + Na]^+^	54–56	+46.431
**2.6**	C_28_H_31_N_3_O_10_	592[M + Na]^+^	70–72	+54.165
**2.7**	C_29_H_33_N_3_O_10_	606[M + Na]^+^	58–60	+47.709
**2.8**	C_30_H_33_N_3_O_10_	618[M + Na]^+^	64–66	+60.244
**2.9**	C_32_H_31_N_3_O_10_	640[M + Na]^+^	68–70	+43.187
**2.10**	C_33_H_33_N_3_O_10_	654[M + Na]^+^	58–60	+47.548
**2.11**	C_32_H_30_ClN_3_O_10_	675[M + Na]^+^	78–80	+36.528
**2.12**	C_33_H_33_N_3_O_10_	654[M + Na]^+^	53–54	+48.155

**Table 2.** Insecticidal activity of triazole derivatives.

Compounds	mortality/% ± SD
**2.1**	0
**2.2**	26 ± 2.42
**2.3**	10 ± 2.42
**2.4**	32 ± 2.37
**2.5**	10 ± 2.42
**2.6**	8 ± 0.00
**2.7**	29 ± 4.15
**2.8**	0
**2.9**	0
**2.10**	0
**2.11**	0
**2.12**	19 ± 2.31
Acetone	0
Phrymarolin-I	43 ± 2.37

Acetone and Phrymarolins I were used as blank and positive controls and the concentration of the tested compounds is 20 ppm.
